# Effects of spinal cord stimulation on heart rate variability in patients with Failed Back Surgery Syndrome

**DOI:** 10.1371/journal.pone.0219076

**Published:** 2019-07-01

**Authors:** Lisa Goudman, Raf Brouns, Bengt Linderoth, Maarten Moens

**Affiliations:** 1 Department of Neurosurgery, Universitair Ziekenhuis Brussel, Brussels, Belgium; 2 Department of Physiotherapy, Human Physiology and Anatomy, Faculty of Physical Education & Physiotherapy, Vrije Universiteit Brussel, Brussels, Belgium; 3 Department of Neurology, ZorgSaam Hospital, Terneuzen, Netherlands; 4 Faculty of Medicine and Pharmacy, Vrije Universiteit Brussel, Brussels, Belgium; 5 Department of Clinical Neuroscience, Karolinska Institute, Stockholm, Sweden; 6 Department of Radiology, Universitair Ziekenhuis Brussel, Brussels, Belgium; 7 Center for Neurosciences (C4N), Vrije Universiteit Brussel, Brussels, Belgium; Université catholique de Louvain, BELGIUM

## Abstract

**Background:**

Building on the recent finding that chronic pain patients with impaired functioning of the descending nociceptive inhibitory system (DNIS) present lower resting heart rate variability (HRV), this study aims to investigate the impact of Spinal Cord Stimulation (SCS) on HRV in patients with Failed Back Surgery Syndrome (FBSS). More precisely, we hypothesize that SCS influences the DNIS, with increased parasympathetic tone as a consequence, as measurable by HRV analysis.

**Methods:**

Twenty-two patients diagnosed with FBSS and treated with SCS participated in this study. HRV was measured with a 2-lead ECG registration tool during on and off states of SCS. HRV analysis for time, frequency, time-frequency and nonlinear domain parameters was based on a 5-minute recording segment.

**Results:**

The mean heart rate and low frequency power were significantly lower when SCS was activated. HRV, absolute and normalized high frequency power significantly increased during SCS compared to without SCS. The ratio of low frequency/high frequency ratios, as parameter for global sympathetic-parasympathetic equilibrium, significantly decreased when SCS was activated.

**Conclusions:**

When SCS is switched off, patients with FBSS present relatively stronger sympathetic tone and weaker parasympathetic activity. Activation of the SCS, possibly via stimulation of the DNIS, restores this disbalance of autonomic activity.

## Introduction

It has previously been suggested that traditional, paresthesia-generating, Spinal Cord Stimulation (SCS) induces several changes in modulation circuits located in the cerebrum and brainstem. An inhibitory effect of traditional SCS on somatosensory evoked potentials, and potential key regions like the thalamus and the anterior cingulate cortex, could play a role in the mechanism of action of SCS as well [1–4]. Several studies provided evidence of the impact of SCS on the descending nociceptive inhibitory system (DNIS) resulting in this inhibitory supraspinal effect [5, 6]. The DNIS comprises a network of cortical and subcortical brain (bilateral anterior insulae, the anterior cingulate cortex, bilateral middle frontal gyri, both amygdalae) and brainstem (rostral ventromedial medulla and the periaqueductal gray) regions that can inhibit nociceptive afferent brain input [7–9] [10, 11]. In several chronic pain syndromes such as knee osteoarthritis, fibromyalgia, painful diabetic neuropathy and low back pain there is an altered functioning of these pathways [12–15].

In healthy subjects, the autonomic nervous system (ANS) is in harmonic balance between the excitatory sympathetic and inhibitory parasympathetic systems. Dysregulation of the ANS has been suggested in chronic pain patients with an overweight of sympathetic activation [16, 17]. This activation is denoted as a factor in pain maintenance and pain itself is a stressor that propagates sympathetic outflow [18].

Heart rate variability (HRV) is the variability in the interval between successive heart beats and is a sensitive predictor of the capacity to regulate emotional responses to threatening intrinsic and extrinsic stressors [19]. HRV has the potential to assess the role of the ANS in normal healthy individuals as well as in patients with chronic diseases [20]. An increased heart rate and reduced HRV has been found in several chronic pain disorders [21–25]. Not only is HRV a plausible modality in the diagnosing process of pain, the use of HRV can also be considered as outcome parameter in measuring the therapeutic effect in chronic pain treatments [26, 27]. Often, studies with HRV describe distinct oscillations, contained in the time interval between successive heart beats, with two primary components; high and low frequency oscillations. High frequency (HF) oscillations can be denoted as vagally mediated, while low frequency oscillations represent a combination of sympathetic and vagal activity [28, 29]. The vagally mediated HF oscillations serve as output measure of the regulatory ability of our brain to control over the periphery of the body [22]. High self-regulatory abilities (i.e. high HF power) are inversely correlated with self-reported symptoms of pain in healthy subjects [23].

Recently, the relationship between the DNIS and HRV has been investigated for chronic pain patients, showing that patients with impaired DNIS presented lower resting HRV [30]. Endogenous analgesia failure (impaired DNIS) is primarily related to the altered function of the parasympathetic system [30].

Up to now, only a very small case series with only 7 patients with chronic pain (among which 2 patients with FBSS) is available, with promising results towards the influence of SCS on the ANS [31]. Therefore, this study aimed to further explore the cardiovascular autonomic modulation among patients with Failed Back Surgery Syndrome (FBSS), with and without activation of their SCS. The cardiovascular autonomic modulation was assessed via analysis of resting HRV, which provides indices of parasympathetic control of heart rate. Accordingly, we hypothesized that SCS influences the DNIS, resulting in an increase of the parasympathetic component, measured by the high frequency power of the resting HRV.

## Method and materials

### Participants

FBSS patients (at least 18 years old) who are treated with SCS at the department of Neurosurgery of Universitair Ziekenhuis Brussel were invited to participate in this study. Patients were excluded if they had one or more coexisting conditions known to affect HRV analysis (including but not limited to atrial fibrillation, numerous atrial or ventricular extra beats, paced rhythm, left ventricular bundle branch block, cancer, kidney or hepatic failure) [31].

**Fig 1:**
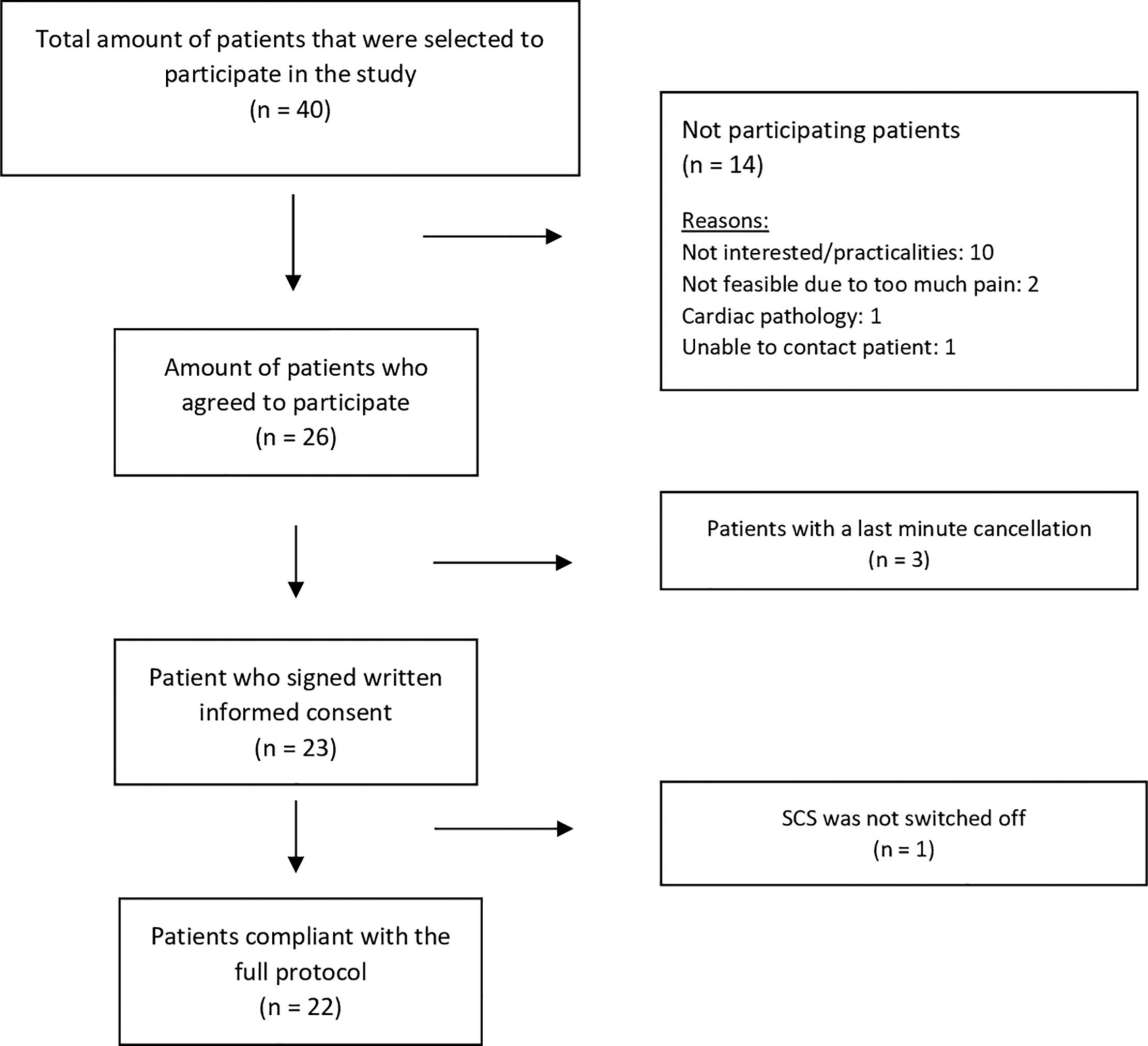
Flow chart of the study.

Of the 40 patients who were contacted to participate, 26 patients agreed to take part in this study. Patient recruitment started on 29/11/2018 and lasted until 21/12/2018. Three patients cancelled their visit on the day of the study visit, wherefore only 23 patients were included. One patient had not switched off the neurostimulator and was therefore excluded, resulting in a total study population of 22 patients. The flow chart of this study is presented in Fig 1.

Six males and 16 females participated in this study with an average age of 55.09 ± 7.63 years. The mean duration that patients were implanted with SCS was 937 ± 648 days. All patients received SCS at level (D8)-D9-D10 and were implanted with either a Senza rechargeable system (Nevro Corp.,Redwood City, CA, USA) with 2*8 contacts or a Restore SensorTM SureScan system connected with a SpecifyTM 5-6-5 SureScan MRI surgical lead (IPG RestoreSensor, Medtronic, Inc., Minneapolis, MN, USA). SCS was delivered with a median charge per pulse of 0.105 (0.06–0.7) μC, median charge per seconds of 550 (49.5–750) μC/sec and a median duty cycle of 30 (25–30) %. Twelve patients supplemented their pain treatment with opioid use and 8 patients were also taking beta-blockers (Table 1).

**Table 1:** Patient characteristics.

Patient	Sex	Age	Opioids	Beta-blockers	VAS back off	VAS back on	VAS leg off	VAS leg on
**A1**	M	57	No	No	8.5	5.2	9.3	3.7
**A2**	F	65	Yes	Yes	10.0	10.0	4.3	9.3
**A3**	M	49	No	Yes	4.1	1.4	4.9	3.6
**A4**	F	72	Yes	Yes	7.8	5.1	7.8	5.4
**A5**	F	37	Yes	No	7.1	5.9	8.9	8.5
**A6**	M	57	No	No	4.3	1.4	4.3	1.5
**A7**	F	44	Yes	No	0.4	0.1	5.7	1.1
**A8**	F	53	No	No	3.8	3.3	6.4	1.4
**A9**	F	54	Yes	Yes	0.0	0.0	0.0	0.0
**A10**	F	60	No	No	6.1	4.1	6.8	3.3
**A11**	F	51	No	No	6.3	5.6	7.0	7.1
**A12**	F	54	Yes	No	9.7	1.3	9.8	5.9
**A13**	F	48	Yes	No	3.7	0.1	5.1	5.4
**A14**	F	57	No	Yes	6.7	6.9	4.6	4.9
**A15**	F	52	No	No	9.8	7.1	3.6	2.7
**A16**	M	46	Yes	Yes	6.9	7.5	6.8	3.3
**A17**	M	55	Yes	No	7.0	6.3	5.4	5.6
**A18**	F	59	No	No	4.3	0.2	3.5	0.1
**A19**	F	57	No	Yes	7.6	3.3	8.6	6.5
**A20**	M	58	Yes	No	5.8	5.2	9.4	7.1
**A21**	F	68	Yes	No	4.8	4.6	2.3	4.9
**A22**	F	59	Yes	Yes	5.3	1.5	4.2	0.4

Abbreviations. F: female, M: male, VAS: visual analogue scale.

The study protocol was approved by the local ethics committee of Universitair Ziekenhuis Brussel (B.U.N. 1423201837785) on 21/11/2018 and registered on clinicaltrials.gov (NCT03768791) on 28/11/2018 (released on 7/12/2018). The authors confirm that all ongoing and related trials for this drug/intervention are registered. All patients provided written informed consent before participation. The study was conducted according to the revised Declaration of Helsinki (1998).

### Study protocol

The study consisted of a single outpatient visit. Patients were asked to switch off their SCS 12 hours before the study visit. All patients were verbally asked to confirm that they switched off SCS 12 hours before the study visit. This statement was also controlled with the aid of the device programming systems. During the study visit, a 5-minute inter-beat (RR) interval measurement was recorded, where after patients were asked to provide a pain intensity score for their back and leg pain. After having filled in the questionnaire, the neurostimulator was switched on again. After a resting period of 40 minutes, RR intervals were registered for 5 minutes and patients again rated their pain intensity. Blinding of patients was not possible, due to the pain relieving effect of SCS that patients felt.

Patients were asked to refrain from alcohol, tobacco, caffeine and drug consumption 24 hours before the study visit [32]. There was no restriction regarding the use of prescribed medication, including analgesics.

### Questionnaire

All participants completed a visual analogue scale (VAS) for assessment of the pain intensity. Pain intensity scores were provided separately for back, left leg and right leg pain. The VAS ranged from no pain to the maximal pain and is expressed in cm from 0 to 10. Patients completed this pain intensity score twice; once after RR registration when SCS was switched off and once after RR registration when SCS was activated. The VAS pain score is broadly accepted as a reliable and valid tool that is sensitive to change [33–35].

### HRV registration and analysis

RR recordings were made with a non-invasive 2-lead ECG registration tool (eMotion HRV sensor (MEGA electronics Ltd., Kuopio, Finland)). The eMotion device is validated for individuals without arrhythmia [36] and has previously successfully been implemented in research settings [37]. The ECG signal was measured by two standard surface electrodes, attached to the patient’s chest. Data was collected at a sampling rate of 1000 Hz/sec and digitally stored on the device. An independent researcher collected all HRV data in all patients.

**Fig 2:**
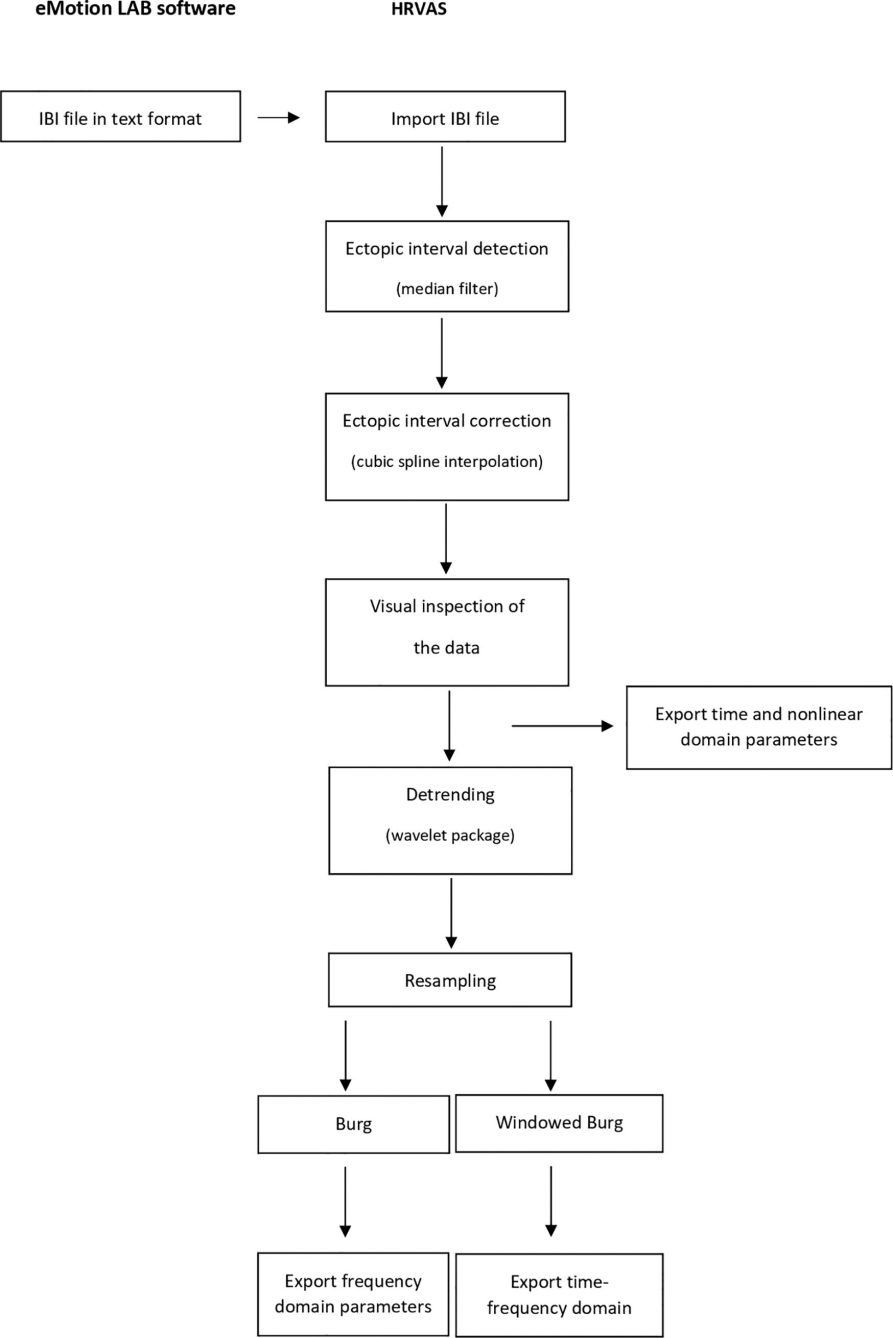
Flowchart HRV preprocessing pipeline.

The ECG signals were saved as text (.txt) files in the eMotion LAB software and a 5 minute segment was selected for off-line analysis using the heart rate variability analysis software (HRVAS) [38]. Preprocessing of the data consisted of ectopic interval detection with a median filter and ectopic interval correction with a cubic spline interpolation method. Detrending, with the wavelet packet technique with a cutoff frequency of 0.0391 Hz [37], and resampling, using interpolation at 2 Hz [39, 40], were performed for analysis in the frequency and time-frequency domain. After preprocessing, data was visually screened for outliers. Suspicious fragments were tested and removed if they met the predefined criteria of an outlier (more than 3 standard deviations, SD). In this study, 0.004% of the recorded data was considered an outlier, probably caused by movement artefacts. The preprocessing pipeline is presented in Fig 2.

HRV analysis was performed in the four major domains being the time, frequency, time-frequency and nonlinear domain. In the time domain mean inter beat interval (IBI), heart rate (HR), standard deviation of normal-to-normal R-R intervals (SDNN), root-mean-square of successive differences of normal-to-normal heart beat interval (RMSSD), HRV triangular index (TI) and triangular interpolation of normal-to-normal heart beat interval histogram (TINN) were calculated.

**Fig 3:**
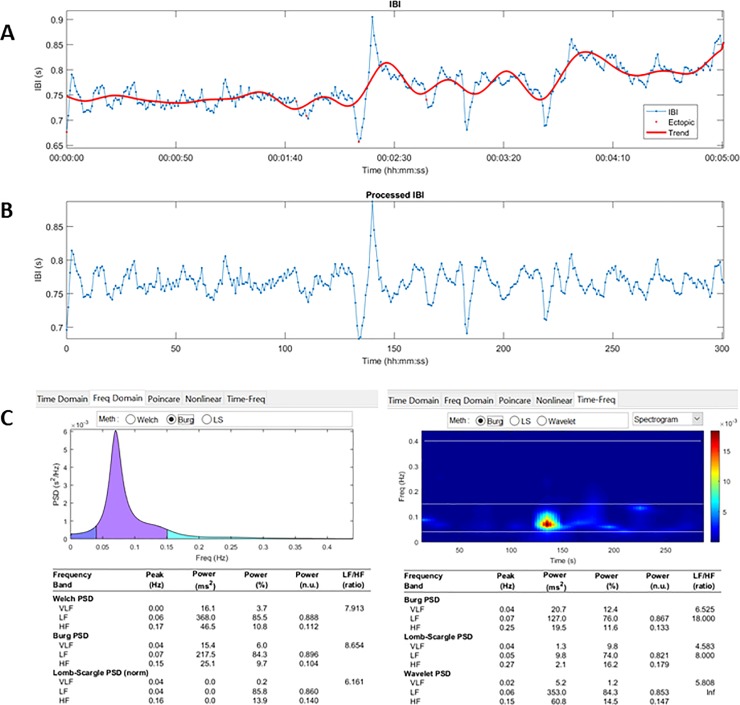
A representative example of HRV data processing. A. unprocessed HRV data. B. preprocessed HRV data (after ectopic interval detection and correction). C. Frequency (left) and time-frequency (right) spectra. Abbreviations. IBI: inter beat interval.

In the frequency domain, spectral power was computed and divided into four frequency bands: ultra low frequency (ULF) from 0 to 0.0033 Hz, very low frequency (VLF) from 0.003 to 0.04 Hz, low frequency (LF) from 0.04 to 0.15 Hz and high frequency (HF) from 0.15 to 0.4 Hz [38]. These frequencies are expressed in absolute numbers. Besides the absolute data, normalized (normalized to total power (LF + HF)) data is provided since they reduce most of the within-and across-subject variability [41]. Power spectrum was calculated with the Burg method [42], an autoregressive spectral estimation procedure, with a model order of 16. Besides power, the ratio of LF to HF (LF/HF) can be calculated in the frequency domain, which provides the sympatho-vagal balance [38]. This measurement is reflecting the relationship between sympathetic and parasympathetic components [43]. This measurement is relying on 4 assumptions namely 1) LF can be primary denoted as sympathetically mediated 2) HF is exclusively represented by parasympathetic activity 3) physiological changes induce reciprocal changes in sympathetic and parasympathetic activity 4) linear relationship between sympathetic and parasympathetic activity on HRV [44]. There is some controversy regarding the fulfilments of the underlying assumptions of this measurement, wherefore this concept has been challenged [44]. One need to be cautious with interpreting the results of this measurement [45]. In Fig 3, an example of the preprocessing and (time-)frequency spectra is provided.

Time-frequency analysis, which enables viewing time and frequency information, was performed with a windowed Burg periodogram (window 30 sec with 15 sec overlap). This procedure consists of resampling the data series and breaking into consecutive segments of equal length [38]. The power spectrum density is then computed for each segment and plotted on a spectrogram with frequency on the y-axis and time on the x-axis. Additionally, the ratio of LF to HF ratios (rLF/HF), i.e. global sympathetic-parasympathetic equilibrium, was calculated. Ratios > 1 are an estimate of sympathetic dominance while ratios <1 are in favour of parasympathetic pre-eminence [37, 38].

Nonlinear techniques such as Poincaré plot analysis (to evaluate self-similarity), sample entropy (to quantify signal complexity) and detrended fluctuation analysis (to assess self-similar properties of non-stationary time series) were also calculated [46, 47]. Poincaré plots are relying on the idea that each IBI is influenced by the preceding IBI [48]. Two measures can be extracted from this type of plot, namely SD1 and SD2. SD1 is based on successive variability between IBIs (“short term variability”) while SD2 is based on the summation of successive IBIs, therefore called “continuous or long term variability” [49]. Sample entropy evaluates the rate of new information that is gathered whereby values of zero mean that sequences are identical to each other and larger values mean a higher complexity [38]. A threshold value of 0.2 was used in combination with a template length of 2 [50]. Detrended fluctuation analysis enables the detection of long-range correlations in time series with a nonstationary character. It provides an output under the form of a scaling exponent alpha, which can be further subdivided into short term scaling (alpha 1) and long term scaling (alpha 2) to provide information about the series self-correlations [51]. DFA was calculated with a break point at 13 beats [38].

### Sample size calculation

Sample size calculation was performed using G*Power 3.1.3 (Düsseldorf, Germany) based on the HF HRV component of a previously reported study in patients with FBSS [31]. Mean HF power components of 70 and 140 msec^2^ were used in the current calculation. The minimal total sample size should reach 24 patients, based on two-tailed testing with alpha = 0.05 and a desired power of 0.95.

### Statistical analysis

All analyses were performed in R Studio version 0.99.903. Normality was controlled with the Shapiro Wilk test and QQ-plots and equality of variances by Levene’s tests. Descriptive statistics are provided as mean (± SD) or as median (interquartile range). Differences in pain intensity scores between the on and off state of SCS were calculated with Wilcoxon tests. HRV data in the four domains between the on and off state was compared with paired t-tests or Wilcoxon tests. P values of 0.05 or less, were considered statistically significant.

## Results

### Descriptive statistics

There was a significant decrease in back pain intensity when SCS was switched on (VAS when SCS off: 6.2 (Q1-Q3: 4.3–7.5); VAS when SCS on: 4.3 (Q1-Q3: 1.4–5.8) (V = 203, p = 0.0003). Leg pain intensity at the symptomatic side revealed a significant decrease when SCS was functioning (off: 5.5 (Q1-Q3: 4.3–7.6), on: 4.3 (Q1-Q3: 1.8–5.8) (V = 190, p = 0.01)).

### HRV parameters

Individual data on HRV parameters can be found in Table 2.

**Table 2:** Individual data on HRV parameters.

Patient	Mean IBI (ms) off	Mean IBI (ms) on	HR (bpm) off	HR (bpm) on	nLF (n.u.) off	nLF (n.u.) on	nHF (n.u.) off	nHF (n.u.) on	rLF/HF off	rLF/HF on
**A1**	866.2	1001.2	70.5	60.2	0.393	0.460	0.607	0.540	0.648	0.853
**A2**	938.7	1062.1	64.0	56.5	0.476	0.277	0.524	0.723	0.909	0.383
**A3**	874.3	845.4	68.7	71.1	0.783	0.630	0.217	0.370	3.614	1.704
**A4**	1137.4	1158.4	52.8	51.9	0.514	0.323	0.486	0.677	1.057	0.477
**A5**	996.4	1098.1	60.4	55.1	0.436	0.149	0.564	0.851	0.772	0.175
**A6**	996.6	767.0	60.3	78.4	0.861	0.867	0.139	0.133	6.180	6.525
**A7**	1082.6	1109.8	55.5	54.2	0.253	0.243	0.747	0.757	0.339	0.322
**A8**	622.1	674.0	98.7	89.1	0.991	0.751	0.009	0.249	105.366	3.011
**A9**	847.3	915.4	71.0	65.6	0.696	0.547	0.304	0.453	2.292	1.206
**A10**	627.1	649.3	95.7	92.4	0.804	0.658	0.196	0.342	4.091	1.922
**A11**	893.8	971.2	67.2	61.9	0.488	0.278	0.512	0.722	0.952	0.385
**A12**	916.1	980.9	65.9	61.2	0.796	0.860	0.204	0.140	3.912	6.136
**A13**	837.0	884.3	72.0	68.0	0.625	0.618	0.375	0.382	1.666	1.619
**A14**	911.4	969.5	65.9	61.9	0.340	0.270	0.660	0.730	0.515	0.370
**A15**	933.1	1062.7	64.4	56.6	0.468	0.322	0.532	0.678	0.880	0.474
**A16**	1030.8	1155.7	58.5	51.4	0.542	0.558	0.458	0.442	1.182	1.263
**A17**	1154.4	1176.4	53.4	51.1	0.983	0.490	0.017	0.510	58.095	0.960
**A18**	697.8	729.2	86.2	82.4	0.654	0.688	0.346	0.312	1.888	2.210
**A19**	959.7	1087.2	62.6	55.3	0.555	0.318	0.445	0.682	1.248	0.466
**A20**	1156.0	1146.0	52.0	52.4	0.477	0.203	0.523	0.797	0.913	0.254
**A21**	649.6	755.5	92.4	82.4	0.688	0.990	0.312	0.010	2.205	97.634
**A22**	893.3	883.2	67.2	68.0	0.627	0.588	0.373	0.412	1.681	1.425

Abbreviations: Bpm: beats per minute, HF: high frequency, HR: heart rate, HRV: heart rate variability, IBI: inter beat interval, LF: low frequency, n.u.: normalized unit, rLF/HF: ratio of LF/HF ratios.

#### Time domain

**Fig 4:**
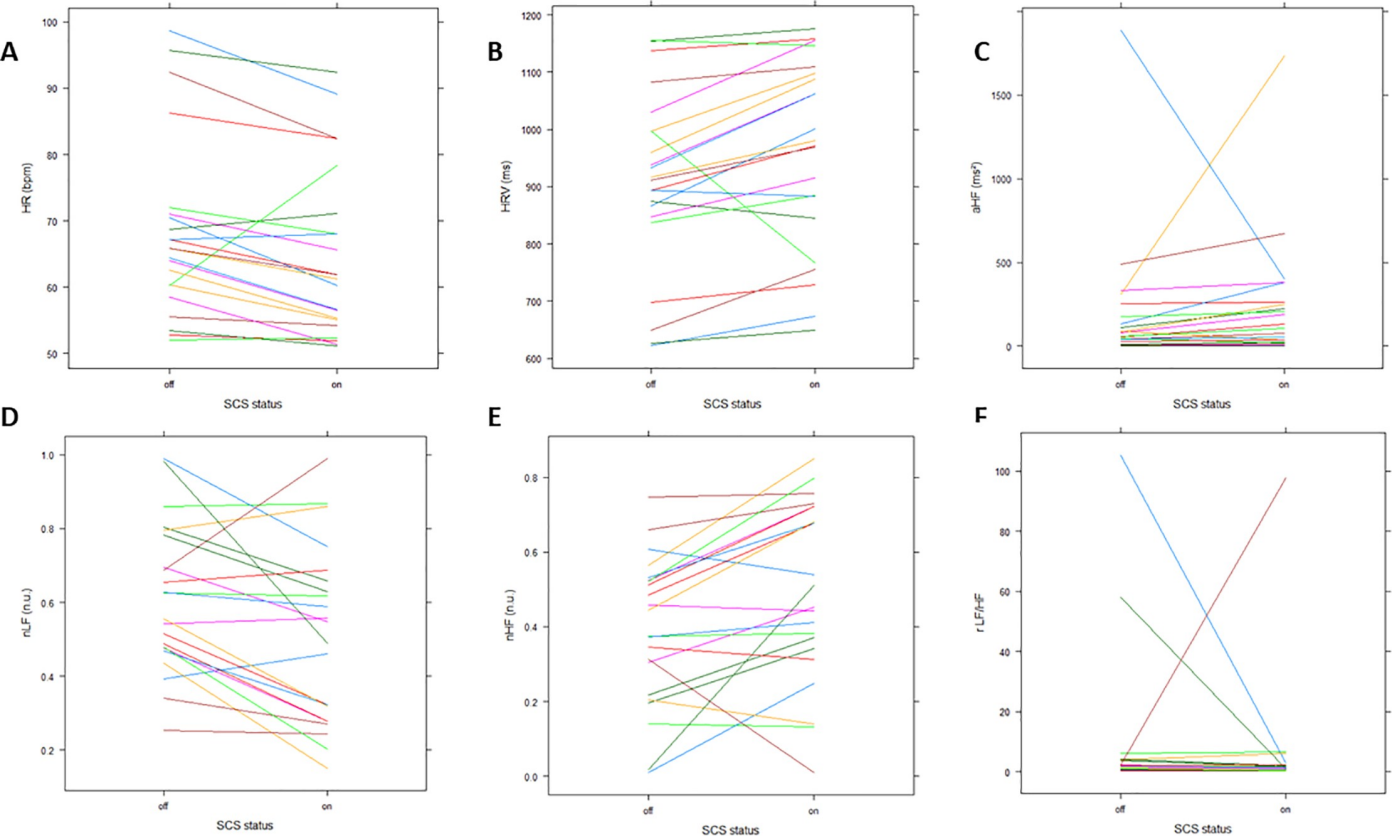
Spaghetti plots of significant results of SCS on HRV parameters in the time and time-frequency domain. All presented plots are representing significant differences between on and off states of SCS in individual FBSS patients. A. HR (p = 0.001); B. HRV (p = 0.001); C. aHF (p = 0.007); D. nLF (p = 0.006); E. nHF (p = 0.006); F. rLF/HF (p = 0.04). Abbreviations. A: absolute, HF: high frequency, HR: heart rate, HRV: heart rate variability, LF: low frequency, rLF/HF: ratio of LF to HF ratios, n: normalized.

The mean HR was significantly lower when SCS was switched on (SCS off: 65.9 beats per minute (bpm) (60.33–70.88); SCS on: 61.55 bpm (55.15–70.33)) (V = 222, p = 0.001) (Fig 4A). There was a significant increase in mean IBI when SCS was switched on (976 ms (854.9–1095.4)) compared to switched off (913.8 ms (852–996.5)) (V = 32, p = 0.001) (Fig 4B). The other parameters for the time domain did not reveal significantly different results when comparing between on and off states of SCS (Table 3).

**Table 3:** Average HRV parameters.

	SCS off	SCS on	P value
**Time domain**			
**Mean IBI (ms)**^a^	913.8 (852–996.5)	976 (854.9–1095.4)	0.001*
**HR (bpm)**^a^	65.9 (60.33–70.88)	61.55 (55.15–70.33)	0.001*
**SDNN (ms)**^a^	41.5 (29.4–63.23)	39.15 (27.12–51.02)	0.75
**RMSSD (ms)**^a^	27.95 (15.03–47.62)	30.75 (16.88–51)	0.54
**TI**^b^	7.44 ± 3.77	8.11 ± 3.18	0.55
**TINN (ms)**^a^	135.7 (82.1–176.6)	136.3 (110.5–168.3)	0.26
**Frequency domain**			
**Absolute LF (ms^2^)**^a^	99.39 (62.40–260.76)	98 (54.36–244.21)	0.40
**Absolute HF (ms^2^)**^a^	66.48 (33.67–163.22)	114.17 (27.5–285.21)	0.01*
**Normalized LF (n.u.)**^b^	0.60 ± 0.20	0.51 ± 0.25	0.02*
**Normalized HF (n.u.)**^b^	0.41 ± 0.20	0.49 ± 0.25	0.02*
**LF/HF**^a^	1.48 (1.03–2.59)	1.10 (0.52–2.39)	0.13
**Time-frequency domain**			
**Absolute LF (ms^2^)**^a^	110.84 (53.60–282.58)	120.36 (51.98–215.29)	0.66
**Absolute HF (ms^2^)**^a^	68.99 (31.86–164.94)	120.43 (26.07–258.32)	0.007*
**Normalized LF (n.u.)**^b^	0.61 ± 0.20	0.52 ± 0.24	0.006*
**Normalized HF (n.u.)**^b^	0.39 ± 0.20	0.50 ± 0.24	0.006*
**rLF/HF**^a^	1.46 (0.91–3.28)	1.08 (0.40–1.87)	0.04*
**Nonlinear analysis**			
**Poincaré SD1**^a^	19.8 (10.62–33.6)	21.75 (11.97–36.15)	0.58
**Poincaré SD2**^a^	56.5 (38.88–82.62)	49.35 (36.45–61.77)	0.54
**Sampen**^a^	1.50 (0.87–1.74)	1.63 (1.20–1.83)	0.22
**DFA alpha**^a^	0.87 (0.78–1.04)	0.91 (0.84–0.99)	0.63
**DFA alpha 1**^b^	1.05 ± 0.42	0.99 ± 0.38	0.41
**DFA alpha 2**^a^	0.87 (0.75–1.03)	0.92 (0.80–0.98)	0.39

Summary of the calculated HRV parameters in the four domains during SCS on and off states.

*: significant result

^a^: Wilcoxon test

^b^: paired t-test.

Abbreviations. Bpm: beats per minute, DFA: detrended fluctuation analysis, HF: high frequency, HR: heart rate, IBI: inter beat interval, LF: low frequency, LF/HF: ratio of LF to HF, n.u.: normalized unit, RMSSD: root-mean-square of successive differences of normal-to-normal heart beat interval, sampen: sample entropy, SDNN: standard deviation of normal-to-normal R-R intervals, TI: HRV triangular index, TINN: triangular interpolation of normal-to-normal heart beat interval histogram.

#### Frequency domain

Normalized LF significantly decreased when SCS was switched on (t(21) = 2.52, p = 0.02), yet absolute LF power was not significantly different between both states (V = 140, p = 0.40). Both absolute and normalized HF power were significantly higher when SCS was switched on: absolute HF power with SCS off: 66.48 ms^**2**^ (33.67–163.22) and SCS on: 114.17 ms^**2**^ (27.5–285.21); (V = 49, p = 0.01). Post hoc analyses through an unpaired Wilcoxon tests revealed that the differences in absolute HF power were not explainable by sex (W = 35, p = 0.36), nor the use of beta-blockers (W = 55, p = 0.97) or opioids (W = 49, p = 0.49). Normalized HF significantly increased with SCS (t(21) = -2.52, p = 0.02). No differences were found in LF/HF between both states (V = 174, p = 0.13) (Table 3).

#### Time-frequency domain

Normalized LF significantly decreased when SCS was switched on compared to switched off (t(21) = 3.04, p = 0.006) (Fig 4D), while absolute LF did not differ (V = 141, p = 0.66). Absolute and normalized HF power increased when SCS was switched on (aHF: V = 46, p = 0.007; nHF: t(21) = -3.04, p = 0.006) (Fig 4C and 4E). The rLF/HF was significantly lower when SCS was switched on (1.08 (0.40–1.87)), compared to switched off (1.46 (0.91–3.28)) (V = 190, p = 0.04) (Fig 4F).

#### Nonlinear analysis

No statistically significant differences could be revealed when comparing SCS on versus SCS off in the nonlinear domain (Table 3).

## Discussion

This study investigates the influence of SCS on various HRV parameters by comparing cardiovascular autonomic modulation between the on and off states of SCS in patients with FBSS. The predefined hypothesis that SCS leads to an increase of the parasympathetic component, which is captured by HF power, can be confirmed by the increase in HF and decrease in LF oscillations when patients activate their SCS.

Besides the well-known spinal segmental mechanisms of SCS, supraspinal loops were already postulated as important contributors to the inhibitory effect of SCS in 1986 [52]. An increase in spinal release of serotonin, a decrease in γ-AminoButyric Acid release in the periaqueductal gray and an activation of the serotonergic system in the rostroventromedial medulla are among the indications for a descending pain modulatory system originating from the brainstem, partly involved in the pain relieving effect of SCS [53–55]. The association between peripheral and central systems regulating cardiovascular function and pain modulatory systems is established through the nucleus tractus solitarius (NTS) by vagal-nociceptive interactions [56, 57]. The NTS receives input from both nervus Vagus and vagal afferents, which allocate the NTS as initial relay for vagally mediated nociceptive effects [56]. Besides the ascending pathways that provide input to the NTS, there is also input from the DNIS in this anatomical region [58]. In terms of autonomic outflow are both sympathetic and parasympathetic preganglionic nuclei receiving input from the DNIS, enabling them to exert an influence on pain thresholds and modifying autonomic outflow [58, 59].

HRV offers a minimally-invasive measurement for the autonomic nervous system activity. HF oscillations are often denoted as a surrogate measure of vagal activity while LF oscillation are considered as a combination of sympathetic and vagal activity [28]. The ratio of low-to-high frequency band power ratios, assesses global sympathetic-parasympathetic balance [29]. In this study, absolute and normalized HF power increased, normalized LF power decreased, as did rLF/HF significantly decreased during SCS. Absolute LF power did not revealed a significant effect induced by SCS. This might be due to the high individual variability, which is reduced by the normalization process [41]. This indicated that there is too much individual variability in absolute LF power to compare between both SCS conditions, emphasizing the role of normalized data. These results are indicative for dominant sympathetic activity when SCS is switched off, with an “under-utilization” of the parasympathetic system. When SCS is functioning, the overweight of the sympathetic system reduces and the parasympathetic system gains more weight [60]. This shift in dominance can be observed through the rLF/HF, as parameter for global sympathetic-parasympathetic balance, which is known to be larger in chronic pain patients compared to healthy controls [19]. The observation of a reduced cardiac sympathetic activity during SCS, is consistent with the findings as seen in patients with chronic refractory angina [60, 61]. Additionally, a meta-analysis in a wide variety of chronic pain disorders revealed a decrease in HF power with a moderate effect estimate, suggestive for a decrease in parasympathetic activation in a chronic pain population, compared to healthy controls [19]. Specifically in patients with chronic low back pain, an increase in normalized LF power and a decrease in normalized HF power was observed, compared to healthy controls [62]. These findings are similar to our results when SCS was not functioning. Based on the current results and the results in other populations, it may be suggested that SCS alters parasympathetic activity in a positive way, also in patients with FBSS. Additionally, an inhibition of sympathetic activity through SCS could be a complementary mechanism, as already suggested in previous studies [63, 64].

Transmission of nociceptive information from the periphery is suppressed by the DNIS and the interface between autonomic and sensory systems [24]. Impaired functioning of the vagal pathway, may correspond to malfunctioning of the descending spinal inhibitory control mechanisms (meaning reduced inhibition of nociceptive information), as seen under the form of central sensitization in chronic pain disorders [24,25,32,50]. HRV can thus be seen as a proxy measure for vagal activity and may by such reflect the functioning of the DNIS [24].

Current literature on this topic is limited to a small case series exploring the effect of SCS on HRV parameters in seven patients with chronic pain, among which two patients with FBSS [31]. A decrease in the HF component was detected during SCS stimulation compared to off states. This result is in strong contrast with the results of this study and with existing literature, as also acknowledged by the authors [31]. The authors explained their results by suggesting that SCS induces different effects in different populations. Our results, however, favor the existence of a major modulation of autonomic activity including an increase in parasympathetic control and a corresponding decrease of sympathetic activity during SCS treatment.

In the time domain, an increased HRV and decreased HR were found when SCS was enabled. Both parameters have an overlapping distribution when comparing these variables during on and off modes of SCS. This demonstrates that both measurements are highly individually determined [45]. SCS is not influencing geometric measures and non-linear parameters in this study, indicating that SCS is only able to influence the standard linear methods and not the more complex measurements with nonlinear components. Higher HRV is believed to reflect greater autonomic flexibility and better regulated emotional responding to threatening situations. Lower variability is a marker for autonomic rigidity, with heightened emotional reactivity to threat [57]. Lower HRV is associated with worse autonomic health and indicative for reduced parasympathetic cardiac control [65]. Additionally, reduced HRV has been associated with the pathogenesis of chronic pain disorders [66]. In a chronic low back pain population, reduced HRV and increased HR were described, suggesting autonomic dysregulation in this population, which is in line with our results [21]. Moreover, reduced HRV as measured by time domain parameters in SCS off states correspond with reduced parasympathetic cardiac control and, are perfectly in line with the results from the (time-) frequency domain. However, previous research revealed that SCS was rather inducing a decrease of sympathetic function than an increase in parasympathetic activity, suggesting a more important contribution of the ascending fibers compared to the descending fibers [63, 64]. Regardless of the dominant contribution of ascending or descending fibers, the output is the same.

HRV holds the potential to objectify pain intensity since an increase in parasympathetic nervous activity, as measured by HRV parameters, may indicate pain relief [27]. The use of HRV as a biomarker for pain relief is a promising approach as it may help overcome the problems related to subjective questionnaires for measuring pain intensity. Especially in this chronic pain population, suboptimal concordance between subjective and objective reporting has been reported [67].

We report the largest cross-over study investigating the effect of SCS on HRV in a population of patients with FBSS. The study was conceived with a very specific working hypothesis in mind, which has been confirmed. Though this is the largest study population reported in literature, we need to acknowledge that the sample size is insufficient to evaluate the effect of confounders or mediators on the relation between SCS and HRV parameters. Future studies are required for confirmation of the beneficiary effects of SCS on HRV in patients with FBSS and also to evaluate the role of potentially influencing factors (e.g. sex, beta-blockers, opioid use, various SCS stimulation paradigms). Additionally, the authors did not correct for multiple testing, which could have resulted in false positive results. When applying the Simes procedure to correct for multiple testing, the normalized LF and HF in the frequency domain and the rLF/HF in the time-frequency domain are not statistically significant anymore [68]. This means that when correcting for multiple testing, the same conclusions can be made namely that SCS induces an increase in HRV and HF and a decrease in LF power. However, rLF/HF is not able to demonstrate this shift in dominance of sympathetic to parasympathetic system. Another possible limitation of this study is that SCS could have induced artefacts in the power spectrum. Nevertheless, no components of tonic stimulation were detected in the ECG data. To limit the possibility that SCS generates artefacts in the power spectrum, future studies could first apply a low-pass filter before downsampling.

## Conclusions

Relying on the assumption that HRV measurements provide information on the sympathetic and parasympathetic system, patients with FBSS present a dominant sympathetic tone and “under-utilization” of the parasympathetic system when SCS is switched off. SCS reduces this dominance of the sympathetic system and increases the parasympathetic influence. Activation of SCS may influence HRV via activation of the DNIS.
